# Applications of Natural Language Processing and Large Language Models for Social Determinants of Health: Protocol for a Systematic Review

**DOI:** 10.2196/66094

**Published:** 2025-01-21

**Authors:** Swati Rajwal, Ziyuan Zhang, Yankai Chen, Hannah Rogers, Abeed Sarker, Yunyu Xiao

**Affiliations:** 1 Department of Computer Science Emory University Atlanta, GA United States; 2 Department of Epidemiology Harvard T.H. Chan School of Public Health Boston, MA United States; 3 Department of Population Health Sciences Weill Cornell Medicine New York, NY United States; 4 Woodruff Health Sciences Center Library Emory University Atlanta, GA United States; 5 Department of Biomedical Informatics Emory University Atlanta, GA United States

**Keywords:** social determinants of health, SDOH, natural language processing, NLP, systematic review protocol, large language models, LLM

## Abstract

**Background:**

In recent years, the intersection of natural language processing (NLP) and public health has opened innovative pathways for investigating social determinants of health (SDOH) in textual datasets. Despite the promise of NLP in the SDOH domain, the literature is dispersed across various disciplines, and there is a need to consolidate existing knowledge, identify knowledge gaps in the literature, and inform future research directions in this emerging field.

**Objective:**

This research protocol describes a systematic review to identify and highlight NLP techniques, including large language models, used for SDOH-related studies.

**Methods:**

A search strategy will be executed across PubMed, Web of Science, IEEE Xplore, Scopus, PsycINFO, HealthSource: Academic Nursing, and ACL Anthology to find studies published in English between 2014 and 2024. Three reviewers (SR, ZZ, and YC) will independently screen the studies to avoid voting bias, and two (AS and YX) additional reviewers will resolve any conflicts during the screening process. We will further screen studies that cited the included studies (forward search). Following the title abstract and full-text screening, the characteristics and main findings of the included studies and resources will be tabulated, visualized, and summarized.

**Results:**

The search strategy was formulated and run across the 7 databases in August 2024. We expect the results to be submitted for peer review publication in early 2025. As of December 2024, the title and abstract screening was underway.

**Conclusions:**

This systematic review aims to provide a comprehensive study of existing research on the application of NLP for various SDOH tasks across multiple textual datasets. By rigorously evaluating the methodologies, tools, and outcomes of eligible studies, the review will identify gaps in current knowledge and suggest directions for future research in the form of specific research questions. The findings will be instrumental in developing more effective NLP models for SDOH, ultimately contributing to improved health outcomes and a better understanding of social determinants in diverse populations.

**International Registered Report Identifier (IRRID):**

DERR1-10.2196/66094

## Introduction

### Rationale

#### Social Determinants of Health

Social determinants of health (SDOH) are the nonmedical conditions in which people are born, grow, live, work, and age [1]. These circumstances are shaped by the distribution of money, power, and resources at global, national, and local levels, and are primarily responsible for health inequities—the unfair and avoidable differences in health status seen within and between countries. As a result, one of Healthy People 2030’s five overarching goals is specifically related to SDOH: “Create social, physical, and economic environments that promote attaining the full potential for health and well-being for all” [2]. Studies have shown that SDOH has powerful influences on health [3] and well-being at the individual and population levels [4]; therefore, it is important to capture them. Such information is valuable to a range of stakeholders including patients, health care providers, policymakers, and insurance companies. For instance, clinicians might modify a patient’s medication regimen if they learn that the patient has difficulty affording their prescriptions or lacks reliable transportation to the pharmacy. It is important to note here that SDOH concepts are often described using a variety of synonyms, paraphrases, or language variations [5], which makes it difficult for simple keyword-based methods to capture all relevant instances. Moreover, many SDOH indicators are not directly stated but implied through context.

#### Natural Language Processing

Natural language processing (NLP) is a subfield of artificial intelligence and computer science that enables computers to understand, interpret, and generate human language in a way that is both meaningful and useful. NLP encompasses a wide range of tasks, including but not limited to language translation, sentiment analysis, speech recognition, text summarization, and information extraction. NLP offers a promising tool to systematically analyze vast amounts of unstructured text data, including electronic health records (EHRs), social media, public health reports, and others [6]. Such tools and techniques can, therefore, be used to extract socioeconomic factors, environmental conditions, and personal lifestyles that significantly impact an individual’s health outcomes. NLP models trained on labeled datasets can learn to recognize these patterns, which can improve extraction accuracy. SDOH information is often embedded within patient narratives or longer descriptions. NLP can assist in parsing and understanding context, coreferences, and the relationships between text parts. Unlike a manual review (time-consuming, labor-intensive, and prone to human error), NLP enables the rapid and scalable analysis of large datasets with greater accuracy and consistency [7,8].

#### NLP for SDOH

Multiple studies exist that showcase the application of NLP to various SDOH-related tasks in addressing health disparities. One key application is the identification and extraction of SDOH factors from EHRs, where NLP techniques have been used to classify and analyze clinical notes for social needs, behavioral factors, and other determinants that influence patient outcomes [9-12]. In the context of public health, NLP has been used to explore the impact of external events (eg, the COVID-19 pandemic) on marginalized communities by analyzing social media data [13]. Additionally, NLP has been leveraged to identify SDOH in specific populations, such as pediatric patients [14,15], patients living with Alzheimer disease [11], and individuals with lower back pain [16]. Advanced models such as the recent GPT and transformer-based models have further refined the extraction of SDOH from patient records and contributed to more precise and scalable analyses [17,18]. Moreover, NLP has been used to find out the underlying social factors in clinical social work notes [19] and emergency medical services records [20] that further enhance the understanding of how social determinants influence health care delivery.

Despite the promise of NLP in the SDOH domain, the literature is dispersed across various disciplines. Hence, a systematic review is necessary to consolidate existing knowledge, identify knowledge gaps in the literature, and inform future research directions in this emerging field. Our systematic review will focus on studies from 2014 onwards which is well-supported by the significant advancements in NLP techniques and the conceptual evolution of SDOH research that have occurred during this period. The development and introduction of Word2Vec [21] and the subsequent introduction of transformer models [22] marked a point of great improvement in NLP research. In addition, the rise in popularity of large language models (LLMs) such as BERT [23] and GPT [24] has driven substantial progress in the field of NLP. By limiting the review to this decade, the systematic review will capture the evolution of NLP approaches and their application in SDOH tasks, thereby providing a comprehensive and up-to-date study of the literature.

### Objectives

This systematic review has two major objectives. First, to identify and highlight distinct NLP techniques, including LLMs (commercial, as well as open-sourced), which are used for tasks including but not limited to augmentation, organization, annotation, prediction, trend analysis, detection, identification, extraction, or classification of SDOH in a given dataset. The NLP model can be used in conjunction with other techniques, but at minimum, the NLP element should be there. The second objective is to report the effectiveness of such techniques or models, identify potential knowledge gaps, and design research questions for future studies.

## Methods

### Protocol and Registration

The study has been registered in PROSPERO (CRD42024578082) and will be carried out under PRISMA (Preferred Reporting Items for Systematic Reviews and Meta-Analyses) guidelines [25]. This protocol was developed using the 2015 PRISMA-P recommended checklist (Multimedia Appendix 1) for systematic review protocols [26]. However, we omit items 16 (meta-biases) and 17 (confidence in cumulative evidence), given we will not synthesize the outcomes of studies. The review team is composed of researchers across disciplines with diverse backgrounds.

### Eligibility Criteria

The eligible publications for this review are restricted to peer-reviewed published literature (observational studies, algorithm validation studies, computational model evaluations, experimental, and qualitative), including journal studies and full conference papers such as ACL anthology. The study must be written in English, although the language of the textual dataset used can vary. The publication period of the included studies was restricted to the last decade, that is, from 2014 to 2024. To be included, a study should answer a research question on the design, development, and application of NLP in health data analysis for SDOH and have used (1) a dataset containing health care data including but not limited to EHRs, social media posts, and clinical notes or narratives, and (2) NLP techniques or models or LLMs (commercial, as well as open source) for tasks including but not limited to augmentation, organization, annotation, prediction, trend analysis, detection, identification, extraction, or classification of SDOH in a given dataset. The NLP model can be used in conjunction with other techniques, but at least the NLP element should be there. Comparisons will be performed against studies included in the review.

For this review, we focused primarily on peer-reviewed literature to ensure the reliability of quantitative information. However, as part of the final systematic review, we will incorporate relevant preprints published between the last search query and up to one month before publication. This approach balances the inclusion of recent advancements while maintaining methodological rigor.

### Information Sources

We conducted a systematic search of the following databases from January 1, 2014, to August 9, 2024, across 7 databases: PubMed, Scopus, Health Source: Nursing/Academic, PsycINFO, Web of Science, ACL Anthology, and IEEE EXPLORE. Preprints (arXiv/bioRxiv), forewords, prefaces, table of contents, programs, schedules, indexes, call for papers or participation, lists of reviewers, lists of tutorial abstracts, invited talks, appendices, session information, obituaries, book reviews, newsletters, lists of proceedings, lifetime achievement awards, erratum, systematic reviews, scoping reviews, and notes will be excluded. We will screen relevant review studies based on eligibility criteria. We will further screen studies that cited the included studies (forward search). We will perform hand searches.

### Search Strategy

The database search strategies were developed by a health sciences librarian (HR) with expertise in literature searches. Known relevant studies collected by the authors were analyzed to select keywords for the search query. The initial search strategy in PubMed was then iteratively developed by adding or removing additional keywords until all known relevant studies were retrieved by the search query. Textbox 1 shows a draft of the search strategy to be used for the PubMed database, including planned limits. The full search strategies for all information sources are provided in Multimedia Appendix 2.

**Search strategy on PubMed.**

**Query**

(“Natural Language Processing”[Mesh] OR “natural language”[tw] OR NLP[tw] OR “large LM*”[tw] OR LLM[tw] OR LLMs[tw] OR “large language model*”[tw] or ChatGPT*[tw] OR “Chat GPT*”[tw] OR GPT4*[tw] OR GPT-4*[tw] OR GPT3*[tw] OR GPR-3*[tw] OR “Generative Pre-trained Transformer*”[tw] OR LLAMA[tw] OR “Claude 3”[tw] OR Mistral[tw] OR MedPaLM*[tw] OR Med-PaLM*[tw] OR “text mining”[tw] OR “text process*”[tw] OR “information retrieval”[tw] OR “information extract*”[tw])

AND

(“Social Determinants of Health”[Mesh] OR SDOH[tw] OR SDH[tw] OR SBDH*[tw] OR “determinants of health”[tw] OR “health determina*”[tw] OR “life events”[tw] OR “social determinant*”[tw] OR “socioeconomic determinant*”[tw] OR “socioeconomic factor*”[tw] OR “social determinate*”[tw] OR “social factor*”[tw] OR “social need*”[tw] OR “social prescribing”[tw] OR “social determining factor*”[tw] OR “social risk*”[tw])

**Filters**

- Manuscript language: English
- Publication years: 2014-2024
- Other: exclude preprints

In addition, a refreshed search strategy will be implemented to ensure that the systematic review includes the most up-to-date and relevant studies. If a significant amount of time has elapsed between the initial literature search and the subsequent stages of citation screening or paper preparation, a secondary search will be conducted. This refreshed search will follow the same search strategy as the initial one, using the same databases, search terms, and inclusion or exclusion criteria. Any newly identified studies will undergo the same rigorous screening process as those identified in the original search to ensure their relevance and quality before inclusion in the final analysis.

### Study Records

#### Data Management

Potentially relevant citations will first be imported into EndNote (Clarivate) and then exported as an XML file to Covidence (Veritas Health Innovation Ltd), which will identify and remove duplicate records.

#### Selection Process

Three reviewers (SR, ZZ, and YC) will independently screen each study for eligibility by marking it as a “yes” (for inclusion), “no” (for exclusion), or “maybe” (in case of uncertainty about relevance) in the Covidence platform. Two senior reviewers (AS and YX) will resolve potential discrepancies during any screening step. All voting is blinded, meaning the colleagues cannot see votes until they have cast their own, and vice versa. In the first stage, the reviewers will screen the titles and abstracts of each study as identified in the databases by our search strategies. In the second stage of screening, the team will obtain and screen the full-text papers as per the eligibility criteria. Studies that do not meet the eligibility criteria will be moved to an exclusion folder. The final corpus of selected studies will be then approved by the consensus of all reviewers involved in this study and sent to an expert consultant for potential suggestions. The selection process will be displayed in a PRISMA flowchart.

#### Data Collection Process

Two independent researchers (SR and ZZ) will extract data from the final included full texts. Before formal data extraction, one reviewer (SR) will pilot the data extraction form with a sample of five papers to identify and address any issues in the form to ensure it is comprehensive. The data extraction will then be conducted independently by both reviewers using the finalized form. Any disagreements between the two reviewers will be discussed and resolved through consensus. If consensus cannot be reached, a third reviewer will mediate. In cases where data is incomplete or unclear, the original study authors may be contacted to request additional information or clarification. All data will be managed using Covidence or via a shared Excel (Microsoft Corp) sheet to ensure consistency throughout the process.

### Data Items

**Figure 1:**
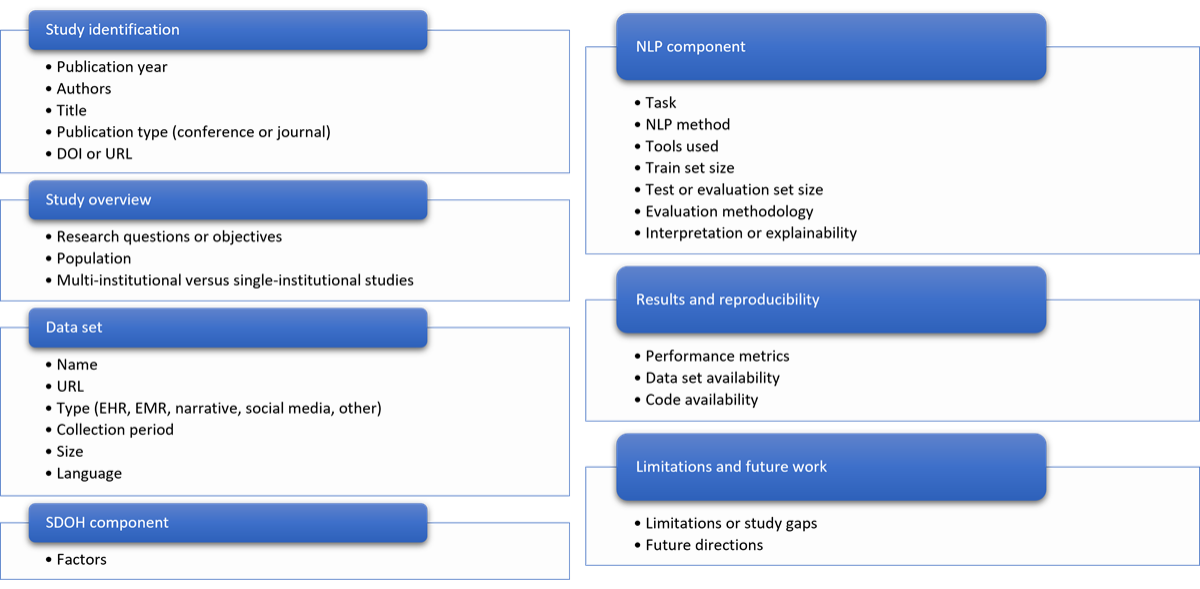
The data to be extracted. EHR: electronic health record; EMR: emergency medical services; NLP: natural language processing; SDOH: social determinants of health.

Figure 1 outlines a preliminary list of variables for which data will be collected, including study identifications (eg, year of publication and type), dataset details (eg, sample size and type), intervention specifics (eg, type of NLP technique and SDOH task), and outcomes (eg, accuracy and precision). Each variable is defined to ensure consistency across studies. During the data extraction process, any assumptions made (such as imputing missing values or converting data into a common format) will be documented and justified. If necessary, modifications to the list of variables will be made during the review, and these changes will be detailed in the final systematic review report.

### Outcomes and Prioritization

We will focus on evaluating the effectiveness and accuracy of various NLP techniques and models applied to SDOH tasks. The primary outcomes of interest will include key performance metrics such as precision, recall, *F*_1_-score, and others, as these are critical indicators of the success of NLP models. Secondary outcomes will involve assessing the integration of NLP techniques with other computational models, such as machine learning and deep learning approaches, to determine their combined impact on model performance. In cases where metrics are not reported, the review will include a qualitative assessment of the study’s findings, focusing on the reported outcomes and their relevance to the SDOH task.

### Risk of Bias Assessment

We anticipate that the studies included in the review may vary significantly. For instance, some may use complex NLP algorithms while others rely on rule-based systems. Hence, it is challenging to name one specific tool for assessing bias at this stage. We will prioritize assessing key factors that could introduce bias, such as the quality of the datasets, the transparency in reporting the models’ performance, and the robustness of the evaluation metrics such as precision and recall.

In addition to assessing general risks of bias, this review will evaluate potential algorithmic bias across individual studies. Algorithmic bias can arise from imbalanced datasets, where certain populations or social determinants are underrepresented, leading to biased predictions. We will examine whether studies report efforts to mitigate such biases.

### Data Synthesis

For this systematic review, a narrative synthesis approach will be used. Data from the included studies will be analyzed by two reviewers, with any disagreements resolved through discussion or consultation with a third reviewer if necessary. Given the diverse methods used in the selected studies, the synthesis will focus on identifying and describing common patterns, trends, and knowledge gaps. The analysis will be divided into the following parts:

- Characteristics of the studies (such as the number of studies, types of datasets, NLP techniques, outcomes, and potential biases).
- Contributions of these studies to the development and application of NLP models for SDOH. As this review is intended to be qualitative, no (or minimum) statistical tools will be applied.

### Ethical Considerations

No ethical approval is required for this protocol and proposed systematic review, as we will only use data from previously published papers that have received ethics clearance and used proper informed consent procedures. The systematic review’s results will be disseminated through publication in a peer-reviewed journal and shared on a publicly accessible GitHub repository [27].

## Results

As of December 2024, the systematic review is in progress (SR, ZZ, and YC started screening studies) and is expected to be finished by March 2025. Our final paper is expected to be submitted to peer-reviewed journals by spring 2025.

## Discussion

### Anticipated Principal Findings

This systematic review is expected to reveal valuable insights into the current state of NLP applications in SDOH tasks. We anticipate identifying the most effective NLP techniques and models across various types of health care data, as well as the common challenges and limitations associated with these approaches. Specifically, we expect to find that the integration of NLP with other computational models, such as machine learning and deep learning, often enhances performance and results in more robust outcomes. We also anticipate that the review will reveal significant variability in model performance based on the type of data used (eg, EHRs vs social media posts) and the specific SDOH being targeted (eg, socioeconomic status vs housing stability). It is important to investigate these factors separately because the challenges in data processing and model effectiveness can vary significantly across different contexts. Investigating these subgroups will provide insights into which NLP techniques are most effective for specific types of data and SDOH, which will enable a more targeted approach in future model development and application. We anticipate that the GitHub repository will offer a platform where researchers can contribute new studies and insights, facilitating ongoing dialogue and collaboration in the field of NLP for SDOH.

### Limitations

One limitation of this study is the selection of studies published between 2014 and 2024. Although this timeframe is chosen to focus on recent advancements, it may limit the historical context. We focus on peer-reviewed papers to ensure the inclusion of high-quality and rigorously evaluated studies. While this approach emphasizes established research, it may mean that some recent studies (such as preprints) will not be captured. By prioritizing peer-reviewed literature, we base our findings on research that has undergone thorough scrutiny.

## Appendix

Multimedia Appendix 1 PRISMA-P 2015 checklist: recommended items to address in a systematic review protocol.

Multimedia Appendix 2 Search strategies and the number of results.

## Abbreviations

EHR: electronic health record
LLM: large language model
NLP: natural language processing
PRISMA: Preferred Reporting Items for Systematic Reviews and Meta-Analyses
PRISMA-P: Preferred Reporting Items for Systematic Reviews and Meta-Analyses—Protocols
SDOH: social determinants of health

## Data Availability

All data generated or analyzed during this study will be included in the final systematic review publication and its supplementary information files.
