# Supplementary material for: Applications of Natural Language Processing and Large Language Models for Social Determinants of Health: Protocol for a Systematic Review
Source: JMIR Res Protoc. 2025 Jan 21;14:e66094. doi: 10.2196/66094 (PMC11795155; doi:10.2196/66094)
Supplement: Multimedia Appendix 2 [file resprot_v14i1e66094_app2.pdf]

Supplementary Document

Index

Search Strategy Results 1
- ACL Anthology 09th August 2024 3
- PubMed 09th August 2024 4
- Web of Science 09th August 2024 5
- IEEE XPLORE 09th August 2024 6
- SCOPUS 09th August 2024 7
- PsycINFO 09th August 2024 8
- Health Source: Nursing/Academic Edition 09th August 2024 9
- ArXiv: 2024-01-28 to 2024-11-28 10

Study Screening Rules 11
Eligibility Criteria 12

Search Strategy Results

S.No.	Database Name	Search Date	Number of Records
1	PubMed	08/09/2024	191
2	Web of Science	08/09/2024	310
3	IEEE Xplore	08/09/2024	1211
4	SCOPUS	08/09/2024	529
5	PsycINFO	08/09/2024	47
6	HealthSource: Academic Nursing	08/09/2024	64
7	ACL Anthology	08/09/2024	51
8	Arxiv	2024-01-28 to 2024-11-28	27

* **1973** Unique records imported into Covidence

ACL Anthology 09th August 2024

Search Results: 52

Note: The records sent to Covidence do NOT include ACL Anthology as there's no way to batch export records. Another team member should go to ACL Anthology and entered this string in the search box:

"Social Determinants of Health" OR SDOH OR SDH OR SBDH* OR “determinants of health” OR “health determina*” OR “life events” OR “social determinant*” OR “socioeconomic determinant*” OR “socioeconomic factor*” OR “social determinate*” OR “social factor*” OR “social need*” OR “social prescribing” OR “social determining factor*” OR “social risk*”

and then downloaded results as appropriate using zotero.

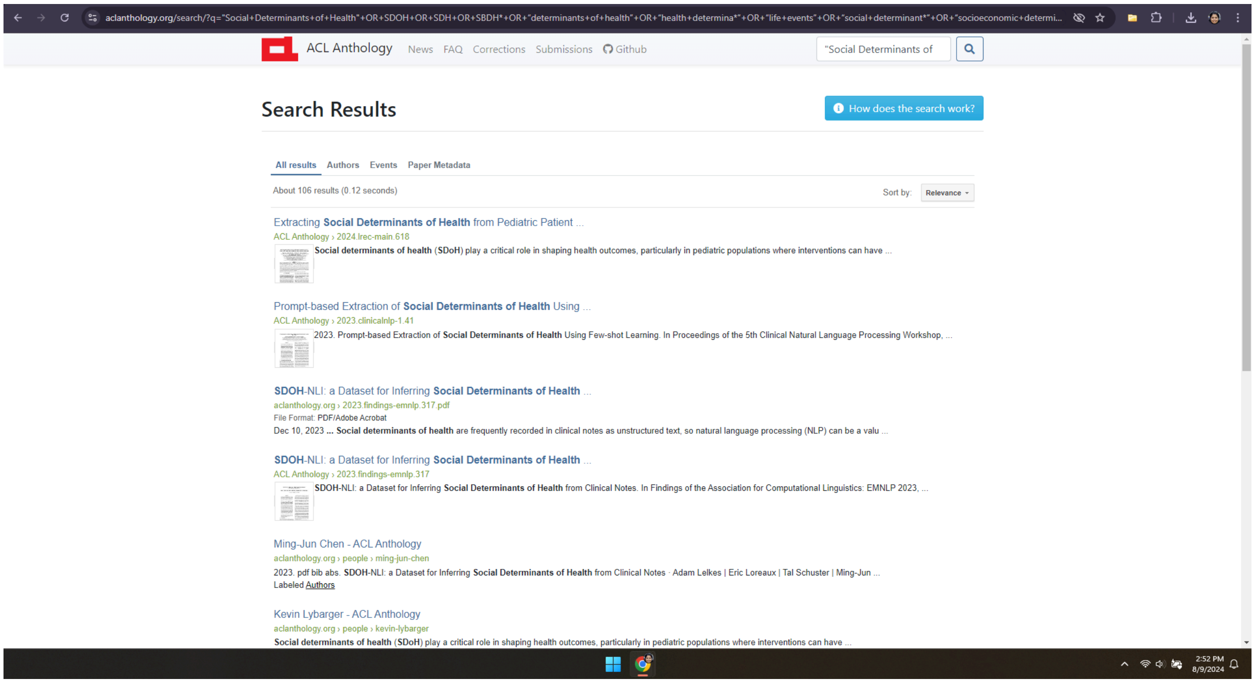

PubMed 09th August 2024

Search Results: 191

Query:

("Natural Language Processing"[Mesh] OR “natural language”[tw] OR NLP[tw] OR “large LM*”[tw] OR LLM[tw] OR LLMs[tw] OR “large language model*”[tw] or ChatGPT*[tw] OR “Chat GPT*”[tw] OR GPT4*[tw] OR GPT-4*[tw] OR GPT3*[tw] OR GPR-3*[tw] OR “Generative Pre-trained Transformer*”[tw] OR LLAMA[tw] OR “Claude 3”[tw] OR Mistral[tw] OR MedPaLM*[tw] OR Med-PaLM*[tw] OR “text mining”[tw] OR “text process*”[tw] OR “information retrieval”[tw] OR “information extract*”[tw])

AND

("Social Determinants of Health"[Mesh] OR SDOH[tw] OR SDH[tw] OR SBDH*[tw] OR “determinants of health”[tw] OR “health determina*”[tw] OR “life events”[tw] OR “social determinant*”[tw] OR “socioeconomic determinant*”[tw] OR “socioeconomic factor*”[tw] OR “social determinate*”[tw] OR “social factor*”[tw] OR “social need*”[tw] OR “social prescribing”[tw] OR “social determining factor*”[tw] OR “social risk*”[tw])

Filters:

- Manuscript language: English
- Publication years: 2014-2024
- Other: Exclude Preprints

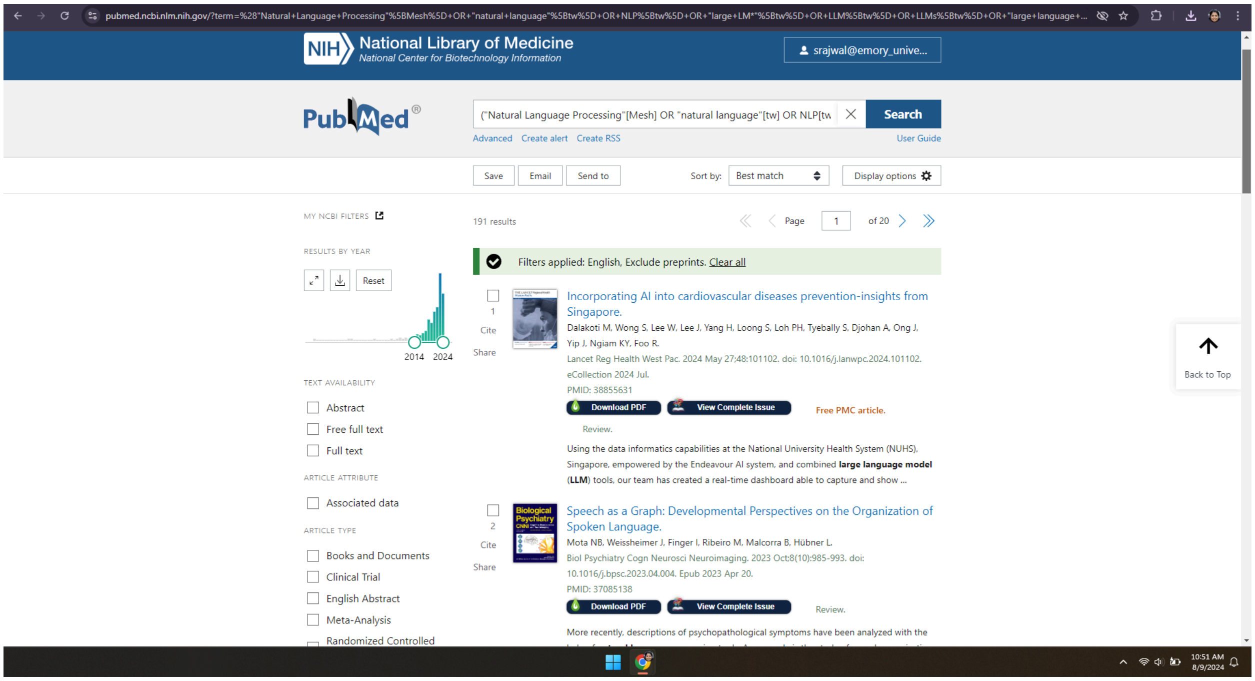

Web of Science 09th August 2024

Search Results: 310

Query:

(“natural language” OR NLP OR “large LM*” OR LLM OR LLMs OR “large language model*” OR ChatGPT* OR “Chat GPT*” OR GPT4* OR GPT-4* OR GPT3* OR GPR-3* OR “Generative Pre-trained Transformer*” OR LLAMA OR “Claude 3” OR Mistral OR MedPaLM* OR Med-PaLM* OR “text mining” OR “text process*” OR “information retrieval” OR “information extract*”)

AND

("Social Determinants of Health" OR SDOH OR SDH OR SBDH* OR “determinants of health” OR “health determina*” OR “life events” OR “social determinant*” OR “socioeconomic determinant*” OR “socioeconomic factor*” OR “social determinate*” OR “social factor*” OR “social need*” OR “social prescribing” OR “social determining factor*” OR “social risk*”)

Filters:

- Manuscript language: English
- Publication years: 2014 to 2024
- Document Types: Article, Proceeding Paper, Early Access, Data Paper

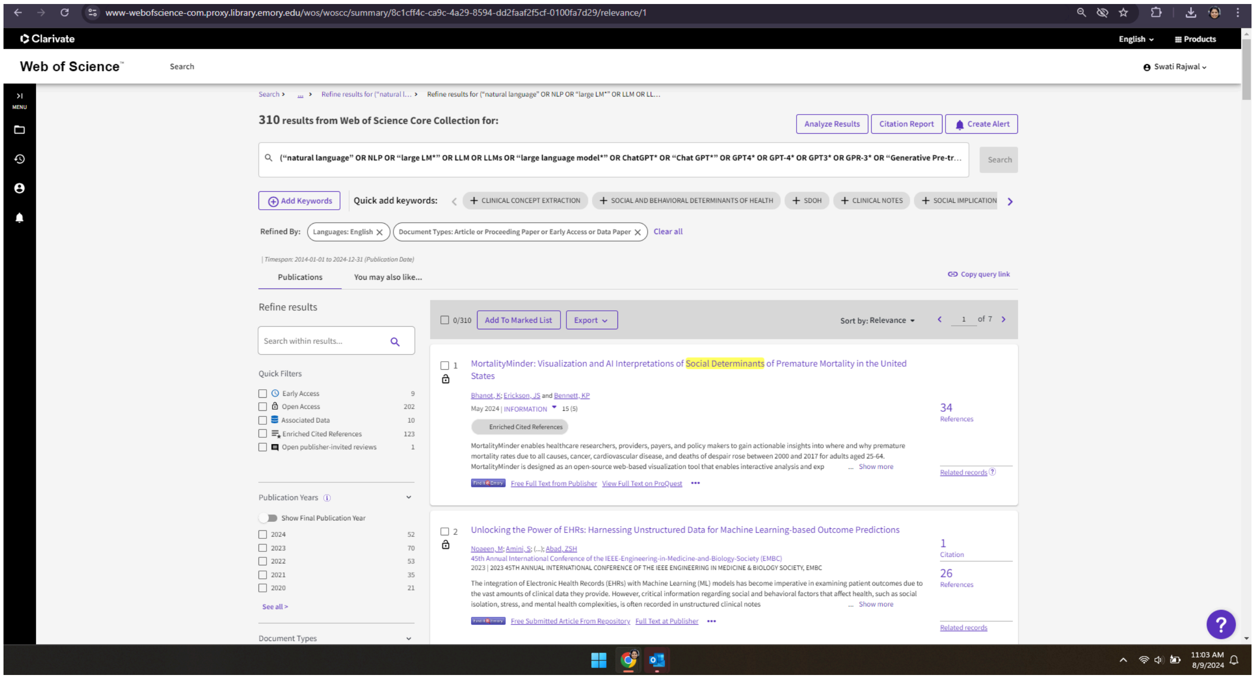

IEEE XPLORE 09th August 2024

Search Results: 1211

Query:

(“natural language” OR NLP OR “large LM” OR LLM OR LLMs OR “large language model” OR “large language models” OR ChatGPT OR “Chat GPT” OR GPT4 OR GPT-4 OR GPT3 OR GPR-3 OR “Generative Pre-trained Transformer” OR LLAMA OR “Claude 3” OR Mistral OR MedPaLM OR Med-PaLM OR “text mining” OR “text process*” OR “information retrieval” OR “information extraction”)

AND

("Social Determinants of Health" OR SDOH OR SDH OR SBDH OR “determinants of health” OR “health determinants” OR “life events” OR “social determinants” OR “socioeconomic determinants” OR “socioeconomic factor*” OR “social determinates” OR “social factor*” OR “social need*” OR “social prescribing” OR “social determining factor*” OR “social risk” OR “social risks”)

Filters

- Publication Years: 2014 to 2024
- Journals

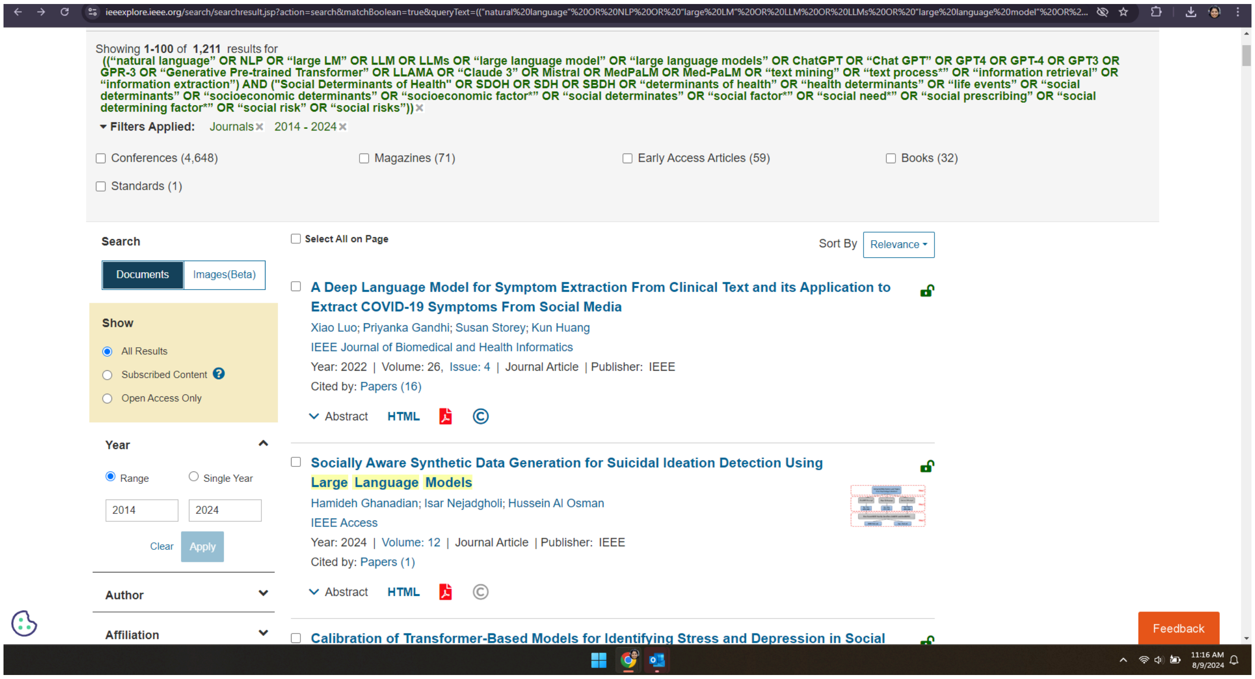

SCOPUS 09th August 2024

Search Results: 529

Query:

(“natural language” OR NLP OR “large LM*” OR LLM OR LLMs OR “large language model*” OR ChatGPT* OR “Chat GPT*” OR GPT4* OR GPT-4* OR GPT3* OR GPR-3* OR “Generative Pre-trained Transformer*” OR LLAMA OR “Claude 3” OR Mistral OR MedPaLM* OR Med-PaLM* OR “text mining” OR “text process*” OR “information retrieval” OR “information extract*”)

AND

("Social Determinants of Health" OR SDOH OR SDH OR SBDH* OR “determinants of health” OR “health determina*” OR “life events” OR “social determinant*” OR “socioeconomic determinant*” OR “socioeconomic factor*” OR “social determinate*” OR “social factor*” OR “social need*” OR “social prescribing” OR “social determining factor*” OR “social risk*”)

Filters:

- 2014-2024
- English
- Article and Conference Paper

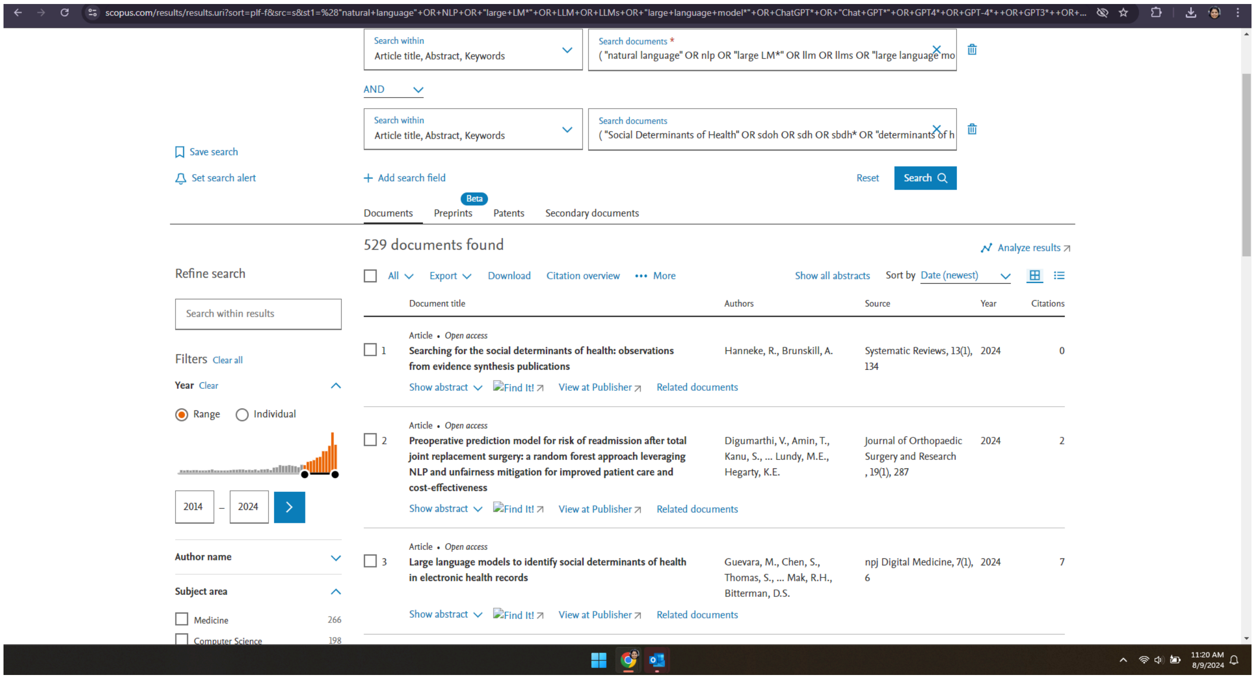

PsycINFO 09th August 2024

Search Results: 47

Query:

(“natural language” OR NLP OR “large LM*” OR LLM OR LLMs OR “large language model*” OR ChatGPT* OR “Chat GPT*” OR GPT4* OR GPT-4* OR GPT3* OR GPR-3* OR “Generative Pre-trained Transformer*” OR LLAMA OR “Claude 3” OR Mistral OR MedPaLM* OR Med-PaLM* OR “text mining” OR “text process*” OR “information retrieval” OR “information extract*”)

AND

("Social Determinants of Health" OR SDOH OR SDH OR SBDH* OR “determinants of health” OR “health determina*” OR “life events” OR “social determinant*” OR “socioeconomic determinant*” OR “socioeconomic factor*” OR “social determinate*” OR “social factor*” OR “social need*” OR “social prescribing” OR “social determining factor*” OR “social risk*”)

Filters:

- 2014-2024
- English
- Academic Journals and Dissertations

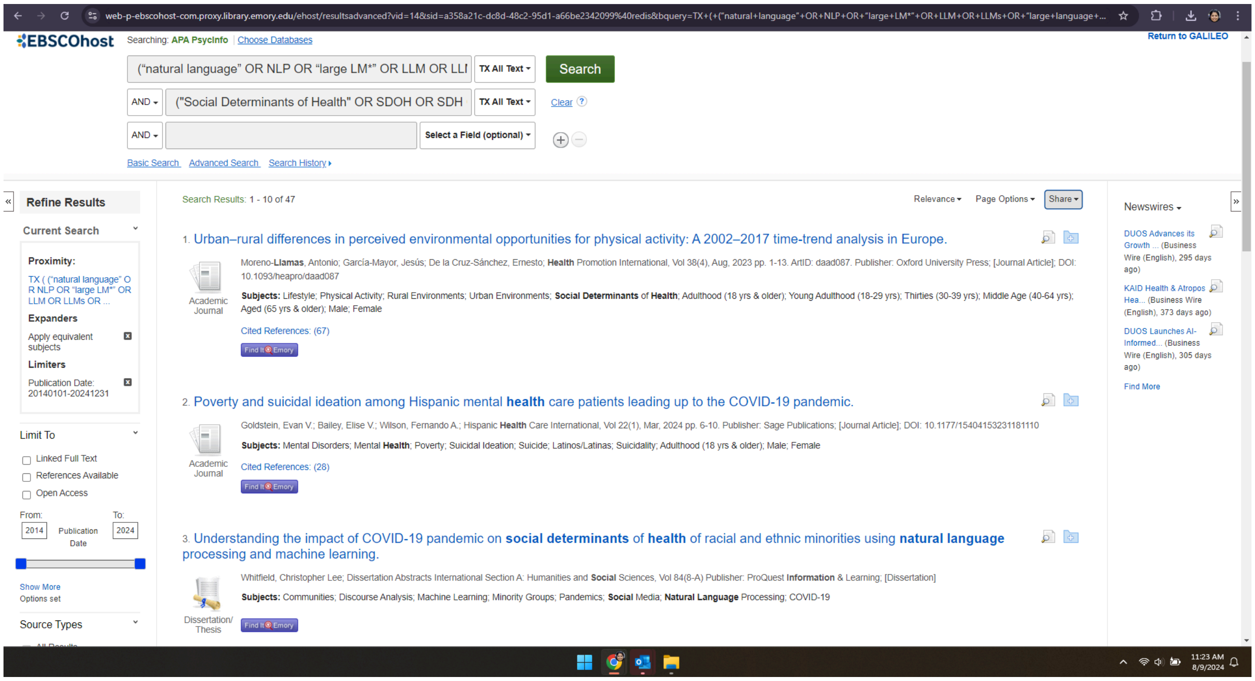

Health Source: Nursing/Academic Edition 09th August 2024

Search Results: 64

Query:

(“natural language” OR NLP OR “large LM*” OR LLM OR LLMs OR “large language model*” OR ChatGPT* OR “Chat GPT*” OR GPT4* OR GPT-4* OR GPT3* OR GPR-3* OR “Generative Pre-trained Transformer*” OR LLAMA OR “Claude 3” OR Mistral OR MedPaLM* OR Med-PaLM* OR “text mining” OR “text process*” OR “information retrieval” OR “information extract*”)

AND

("Social Determinants of Health" OR SDOH OR SDH OR SBDH* OR “determinants of health” OR “health determina*” OR “life events” OR “social determinant*” OR “socioeconomic determinant*” OR “socioeconomic factor*” OR “social determinate*” OR “social factor*” OR “social need*” OR “social prescribing” OR “social determining factor*” OR “social risk*”)

Filters:

- Publication Years: 2014-2024
- Manusript Language: English
- Limit to: Peer-reviewed scholarly journals

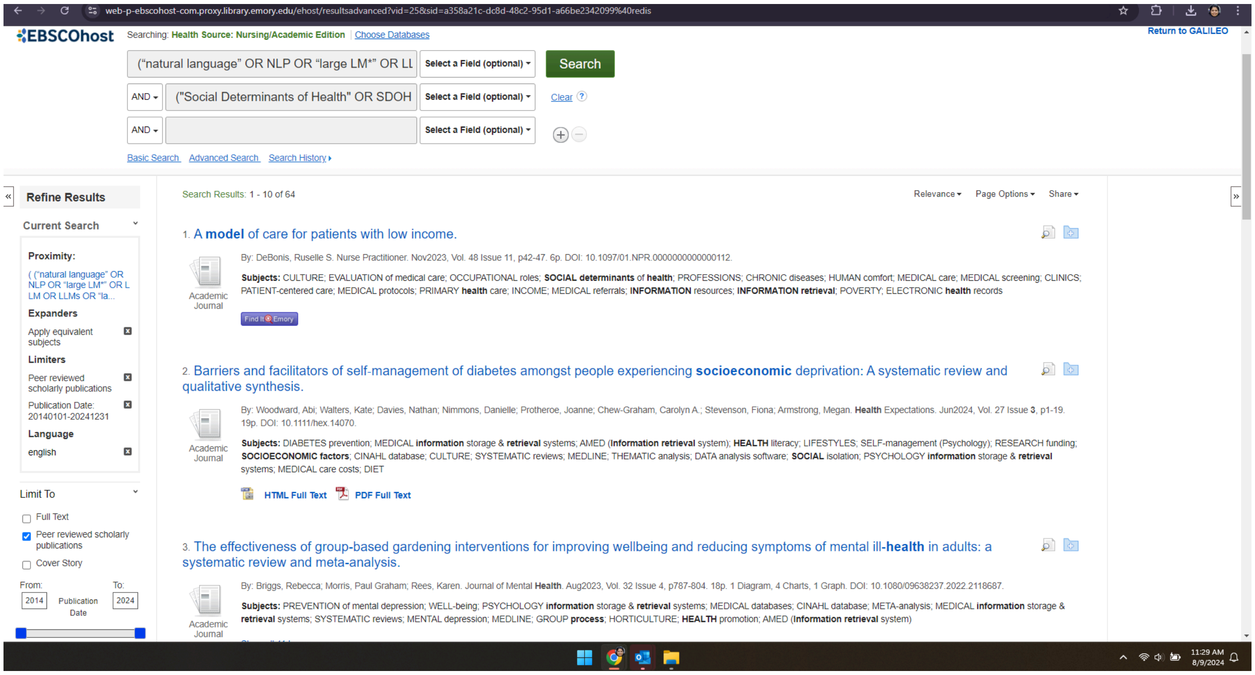

ArXiv: 2024-01-28 to 2024-11-28

Search Results: 27

Query: order: -announced_date_first; size: 200; hide_abstracts: True; date_range: from 2024-01-28 to 2024-11-28; include_cross_list: True; terms: AND all="natural language" OR NLP OR "large LM" OR LLM OR LLMs OR "large language model" OR "large language models" OR ChatGPT OR "Chat GPT" OR GPT4 OR GPT-4 OR GPT3 OR GPT-3 OR "Generative Pre-trained Transformer" OR LLAMA OR "Claude 3" OR Mistral OR MedPaLM OR Med-PaLM OR "text mining" OR "text process*" OR "information retrieval" OR "information extraction"; AND all="Social Determinants of Health" OR SDOH OR SDH OR SBDH OR "determinants of health" OR "health determinants" OR "life events" OR "social determinants" OR "socioeconomic determinants" OR "socioeconomic factor*" OR "social determinates" OR "social factor*" OR "social need*" OR "social prescribing" OR "social determining factor*" OR "social risk" OR "social risks"

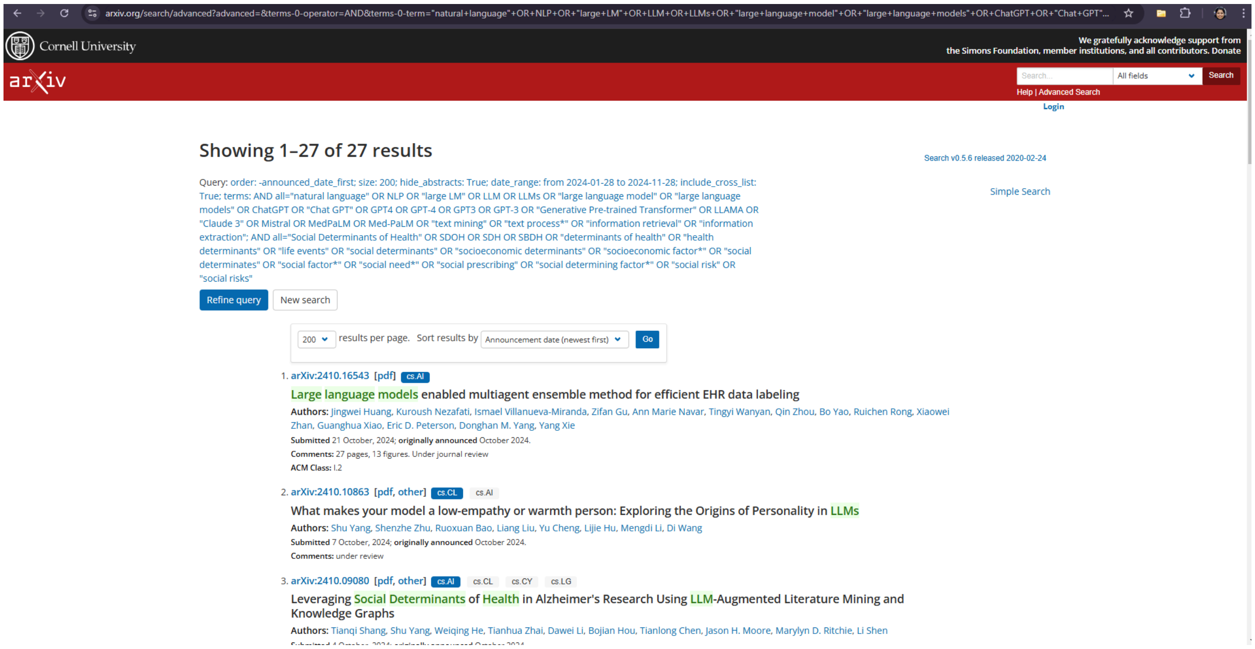
Showing 1–27 of 27 results

Query: order: -announced_date_first; size: 200; hide_abstracts: True; date_range: from 2024-01-28 to 2024-11-28; include_cross_list: True; terms: AND all="natural language" OR NLP OR "large LM" OR LLM OR LLMs OR "large language model" OR "large language models" OR ChatGPT OR "Chat GPT" OR GPT4 OR GPT-4 OR GPT3 OR GPT-3 OR "Generative Pre-trained Transformer" OR LLAMA OR "Claude 3" OR Mistral OR MedPaLM OR Med-PaLM OR "text mining" OR "text process*" OR "information retrieval" OR "information extraction"; AND all="Social Determinants of Health" OR SDOH OR SDH OR SBDH OR "determinants of health" OR "health determinants" OR "life events" OR "social determinants" OR "socioeconomic determinants" OR "socioeconomic factor*" OR "social determinates" OR "social factor*" OR "social need*" OR "social prescribing" OR "social determining factor*" OR "social risk" OR "social risks"

1. arXiv:2410.16543 [pdf] cs.AI
Large language models enabled multiagent ensemble method for efficient EHR data labeling
Authors: Jingwei Huang, Kuroush Nezafati, Ismael Villanueva-Miranda, Zifan Gu, Ann Marie Navar, Tingyi Wanyan, Qin Zhou, Bo Yao, Ruichen Rong, Xiaowei Zhan, Guanghua Xiao, Eric D. Peterson, Donghan M. Yang, Yang Xie
Submitted 21 October, 2024; originally announced October 2024.
Comments: 27 pages, 13 figures. Under journal review
ACM Class: I.2

2. arXiv:2410.10863 [pdf, other] cs.CL cs.AI
What makes your model a low-empathy or warmth person: Exploring the Origins of Personality in LLMs
Authors: Shu Yang, Shenzhe Zhu, Ruoxuan Bao, Liang Liu, Yu Cheng, Lijie Hu, Mengdi Li, Di Wang
Submitted 7 October, 2024; originally announced October 2024.
Comments: under review

3. arXiv:2410.09080 [pdf, other] cs.AI cs.CL cs.CY cs.LG
Leveraging Social Determinants of Health in Alzheimer's Research Using LLM-Augmented Literature Mining and Knowledge Graphs
Authors: Tianqi Shang, Shu Yang, Weiqing He, Tianhua Zhai, Dawei Li, Bojian Hou, Tianlong Chen, Jason H. Moore, Marylyn D. Ritchie, Li Shen

Study Screening Rules

1. The study was registered on PROSPERO 2024 CRD42024578082
2. HR created search queries across PubMed, Web of Science, IEEE Xplore, SCOPUS, PsycINFO, HealthSource: Academic Nursing, and ACL anthology after discussions with the team and created a Covidence group.
3. SR manually added ACL anthology search results since there was no way to batch export records.
4. SR, ZZ, and YC reviewed the screening studies independently on covidence.
5. AS and YX were tagged on covidence in case of conflict to resolve reviewer disagreements.
6. Reviewers followed the eligibility criteria to screen studies.

Eligibility Criteria

PICO (TT)	Inclusion Criteria	Exclusion Criteria
Population	Datasets containing healthcare data including but not limited to electronic health records, social media posts, and clinical notes/narratives.	Datasets not related to healthcare or lacking information on social determinants of health
Intervention	NLP techniques/models/LLMs (commercial as well as open source) are used for tasks including but not limited to augmentation, organization, annotation, prediction, trend analysis, detection, identification, extraction, or classification of social determinants of health in a given dataset. The NLP model can be used in conjunction with other techniques, but at minimum, the NLP element should be there.	Non-NLP techniques or models, or NLP methods not applied to social determinants of health
Comparison	● In NLP, there might not be many studies that do comparison. Rather, most studies introduce their own NLP-based pipeline for SDoH tasks<br>● Comparison can be (and will be) done against studies included in the SLR.<br>● If any exist, studies that compare different NLP models or techniques, or compare NLP methods to manual review or existing benchmarks	
Outcomes	● Effectiveness and accuracy of NLP techniques/models; metrics including but not limited to precision, recall, F1 score<br>● NLP techniques can be applied in conjunction with other models/techniques (such as Machine learning or deep learning models)<br>● In case evaluation metrics are not reported, the review will provide a	Studies that do not (directly/in conjunction with other techniques) use NLP models/techniques for tasks including but not limited to augmentation, organization, annotation, prediction, trend analysis, detection, identification, extraction, or classification SDoH.

	comprehensive summary of the findings from each study.	
Type of Study	Observational studies, algorithm validation studies, computational model evaluations, experimental, qualitative, peer-reviewed & published studies	● Preprints (arxiv/biorxiv)<br>● Forewords, prefaces, table of contents, programs, schedules, indexes, calls for papers/participation, lists of reviewers, lists of tutorial abstracts, invited talks, appendices, session information, obituaries, book reviews, newsletters, lists of proceedings, lifetime achievement awards, erratum, systematic reviews, scoping reviews and notes)
Type of Question	Research questions pertaining to the design, development, and application of NLP in health data analysis for SDoH	Questions unrelated to the methodological application of NLP
Human and/or Animal Studies	N/A	N/A
Publication Type	Peer-reviewed published literature, including journal articles and full conference papers such as ACL anthology.	"Grey" literature such as conference abstracts, posters, and non-peer-reviewed articles
Date range to search	2014-present (last 10 years review)	Publications before the specified date range
Languages to include	English	Non-English
